# Estimation of FBMC/OQAM Fading Channels Using Dual Kalman Filters

**DOI:** 10.1155/2014/586403

**Published:** 2014-02-18

**Authors:** Mahmoud Aldababseh, Ali Jamoos

**Affiliations:** Department of Electronic Engineering, Al-Quds University, P.O. Box 20002, Jerusalem, Palestine

## Abstract

We address the problem of estimating time-varying fading channels in filter bank multicarrier (FBMC/OQAM) wireless systems based on pilot symbols. The standard solution to this problem is the least square (LS) estimator or the minimum mean square error (MMSE) estimator with possible adaptive implementation using recursive least square (RLS) algorithm or least mean square (LMS) algorithm. However, these adaptive filters cannot well-exploit fading channel statistics. To take advantage of fading channel statistics, the time evolution of the fading channel is modeled by an autoregressive process and tracked by Kalman filter. Nevertheless, this requires the autoregressive parameters which are usually unknown. Thus, we propose to jointly estimate the FBMC/OQAM fading channels and their autoregressive parameters based on dual optimal Kalman filters. Once the fading channel coefficients at pilot symbol positions are estimated by the proposed method, the fading channel coefficients at data symbol positions are then estimated by using some interpolation methods such as linear, spline, or low-pass interpolation. The comparative simulation study we carried out with existing techniques confirms the effectiveness of the proposed method.

## 1. Introduction

Wireless multicarrier (MC) communication systems are parallel data transmission techniques in which high data rates can be achieved by the simultaneous transmission over many orthogonal subcarriers [1]. Using MC communications, a wide-band frequency-selective fading channel is divided into many narrow-band frequency nonselective flat fading subchannels, facilitating channel estimation and equalization as well as time synchronization and narrow-band interference mitigation. In addition, the division of the whole bandwidth into many subchannels provides scalability and flexibility when configuring the communication link.

The most widely used multicarrier modulation technique is orthogonal frequency division multiplexing (OFDM) with cyclic prefix (CP) [2]. Due to the various advantages of OFDM, it has become the key physical layer transmission technology adopted in current broadband communication systems such as wireless local area networks (WLAN), worldwide interoperability for microwave access (WiMAX), and long term evolution (LTE) as well as in digital video and audio broadcasting (DVB and DAB) [2, 3]. The OFDM system offers simple implementation using the inverse fast Fourier transforms (IFFT) and the fast Fourier transform (FFT) pairs in the modulator and demodulator, respectively. However, the large side lobes of the frequency response of the filters that characterize the subcarrier channels result in significant interference among subcarriers. As an alternative to OFDM, filter bank multicarrier (FBMC) modulation, which is also implemented based on IFFT/FFT pairs, has several advantages over OFDM [4, 5]. Firstly, it does not require CP resulting in better use of the allocated spectrum. Secondly, instead of using a rectangular window, a prototype filter designed with the Nyquist pulse shaping principle is adopted, which can reduce greatly the spectral leakage problem of OFDM. This results in negligible intersymbol interference (ISI) and intercarrier interference (ICI). Thirdly, the combination of filter banks with offset quadrature amplitude modulation (FBMC/OQAM), known also as OFDM/OQAM, leads to a maximum data transmission rate [6].

Fading channel estimation and equalization techniques are crucial for coherent symbol detection at the receiver. In the framework of multicarrier systems, many channel estimation techniques were proposed in the literature particularly for OFDM systems [7–11]. They can be classified as training sequence/pilot symbols based techniques and blind methods. In this paper, as blind techniques require a large amount of data and have higher complexity than training/pilot based techniques, we will focus our attention on the latter techniques.

Compared to OFDM, few FBMC/OQAM studies have addressed the problem of channel estimation and equalization [12–18]. Indeed, when classical channel estimation methods used for OFDM systems are applied directly to FBMC/OQAM system, an intrinsic intersymbol-interference is observed. This results in system performance degradation. To reduce this intrinsic interference, several methods are recently proposed, including both preamble-based [12–14] and scattered pilots-based [15, 16] channel estimators. In [17], a two-stage minimum mean square error (MMSE) equalizer is proposed to cope more efficiently with intercarrier interference (ICI). A fractionally spaced adaptive decision feedback equalizer (DFE) based on the least mean square (LMS) algorithm was proposed in [18] where the input of each equalizer comprises only the output of each subcarrier.

When dealing with Kalman filter based channel estimators, several approaches were developed for multicarrier systems such as multicarrier code division multiple access (MC-CDMA) and OFDM [10, 11, 19]. Indeed, the Kalman filter combined with an autoregressive (AR) model to describe the time evolution of the fading channel is shown to track channel variations and to provide superior bit error rate (BER) performance over the standard LMS and recursive least square (RLS) estimators [19]. Nevertheless, the AR parameters are unknown and should be estimated. To jointly estimate the autoregressive process and its parameters from noisy observations, one of the authors of this paper has recently proposed dual Kalman filters based structure [11, 20]. This structure consists of two cross-coupled Kalman filters where the first filter uses the latest estimated AR parameters to estimate the fading process, while the second filter uses the estimated fading process to update the AR parameters. In this paper, we propose to investigate the relevance of the dual Kalman filters for the estimation of FBMC/OQAM time-varying fading channels based on pilot symbols. Once the fading channel coefficients at pilot symbol positions are estimated by the proposed method, the fading channel coefficients at data symbol positions are then to be estimated by using some interpolation methods such as linear, spline, or low-pass interpolation.

The remainder of the paper is organized as follows. In Section 2, we explain the FBMC/OQAM system model. The estimation of the fading channels based on dual Kalman filters is introduced in Section 3. Simulation results are reported in Section 4. Conclusion remarks are drawn in Section 5.

## 2. FBMC/OQAM System Model

There are different filter bank multicarrier structures that are proposed in the literature. Among them are discrete wavelet multitone [21], filtered multitone (FMT) [22], cosine modulated multitone (CMT) [23], modified discrete Fourier transform (MDFT) [24], exponentially modulated filter bank (EMFB) [25], and FBMC/OQAM [6]. The last is the most popular filter bank scheme that results in high bandwidth efficiency.

**Figure 1:**
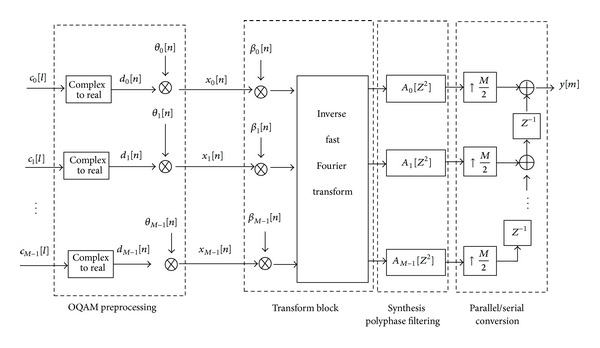
Block diagram of the FBMC/OQAM transmitter.

**Figure 2:**
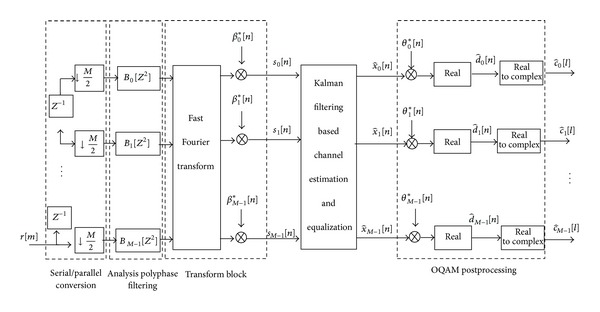
Block diagram of the FBMC/OQAM receiver.

The fundamental parts of the FBMC/OQAM system are the synthesis filter bank (SFB) at the transmitter and the analysis filter bank (AFB) at the receiver, arranged in the so-called transmultiplexer (TMUX) configuration as shown in Figures 1 and 2. It should be noted that the SFB and AFB pairs can be efficiently implemented using FFT and IFFT of size *M* aided by polyphase filtering structures. When using uniformly modulated filter banks [26], a prototype filter *p*[*m*] of length *L*_*p*_ is shifted in frequency to generate subchannels which cover the whole system bandwidth. Thus, the discrete-time baseband signal at the output of the FBMC transmitter with OQAM modulation can be expressed as

(1) $$\begin{array}{c} y\left[m\right]=\overset{M-1}{\underset{k=0}{\sum }}\overset{\infty }{\underset{n=-\infty }{\sum }}{x_{k}\left[n\right]\beta }_{k}\left[n\right]p\left[m-\frac{nM}{2}\right]e^{j(2\pi /M)km}, \end{array}$$

where *m* is the sample index at SFB output/AFB input, *n* is the sample index at OQAM preprocessing output/postprocessing input, *M* is the number of subcarriers in the filter bank, and

(2) $$\begin{array}{c} \beta_{k}\left[n\right]={\left(-1\right)}^{kn}\exp\left(-j\frac{2\pi k}{M}\left(\frac{L_{p}-1}{2}\right)\right), \end{array}$$

(3) $$\begin{array}{c} x_{k}\left[n\right]=d_{k}\left[n\right]\theta_{k}\left[n\right], \end{array}$$

(4) $$\begin{array}{c} \theta_{k}\left[n\right]=j^{k+n}. \end{array}$$

In (3), *d*_*k*_[*n*] are real-valued data symbols at subcarrier *k*, transmitted at a rate of 2/*T* where the signaling period is defined as *T* = 1/Δ*f* with Δ*f* being the subcarrier spacing. The pair of symbols *d*_*k*_[*n*] and *d*_*k*_[*n* + 1] represent, respectively, the in-phase and quadrature parts of the complex-valued input symbol *c*_*k*_[*l*]. This complex-to-real conversion operation is considered to introduce an up-sampling by two. The inverse operation of real-to-complex conversion is performed at the receiver and results in down-sampling the signal by two. Thus, the complex-to-real operation performs the following mapping:

(5) dk[n]={Re(ck[l]),k  evenIm⁡(ck[l]),k  odddk[n+1]={Im⁡(ck[l]),k  evenRe(ck[l]),k  odd,

where *l* is the sample index at OQAM preprocessing input/postprocessing output. Note that the complex-valued input symbols *c*_*k*_[*l*] belong to QAM alphabet with the real and imaginary parts being interleaved by a time offset of *T*/2, resulting in OQAM modulation. To maintain orthogonality, *d*_*k*_[*n*] is multiplied by *θ*_*k*_[*n*].

In order to achieve high spectral efficiency, complex modulated filter banks are usually used, which means that all subchannel filters are frequency shifted versions of the prototype filter *p*[*m*]. So, the *k*th synthesis filter *g*_*k*_[*m*] can be expressed as

(6) $$\begin{array}{c} g_{k}\left[m\right]=p\left[m\right]\exp\left(j\frac{2\pi k}{M}\left(m-\frac{L_{p}-1}{2}\right)\right), \end{array}$$

where *m* = 0,1,…, *L*_*p*_ − 1. The length *L*_*p*_ of the prototype filter *p*[*m*] depends on the size of the filter bank and the number of FBMC/OQAM symbol waveforms *K* that overlap in time as *L*_*p*_ = *KM*.

The *k*th analysis filter *f*_*k*_[*m*] is simply a time-reversed and complex-conjugated version of the corresponding synthesis filter:

(7) $$\begin{array}{c} f_{k}\left[m\right]=g_{k}^{*}\left[L_{p}-1-m\right]. \end{array}$$

It should be noted that the design of the prototype filter must satisfy perfect reconstruction (PR) conditions or at least provide nearly perfect reconstruction (NPR) characteristics. However, the PR property is only achieved under the condition of ideal transmission channel. As interferences in the wireless channel are unavoidable, there is no way to have PR conditions. Thus, prototype filters are designed to satisfy NPR characteristics.

In this paper, we used frequency sampling technique [27] or windowing based approach [28] to design NPR prototype filter. In these methods, the prototype filter coefficients can be obtained using a closed-form representation that includes only a few adjustable design parameters. The impulse response coefficients of the filter are obtained according to the desired frequency response, which is sampled on a *KM* uniformly spaced frequency point *ω*_*k*_ = 2*πk*/*KM*. So, the finite impulse response (FIR) of the low-pass prototype filter can be expressed as [29]

(8) p[m]=P[0] +2∑k=1U(−1)kP[k]cos⁡⁡(2πkKM(m+KM−(Lp−1)2)),

where *m* = 0,1,…, *L*_*p*_ − 1, *P*[0] = 1, and *P*[*c*]^2^ + *P*[*K* − *c*]^2^ = 1 for *c* = 1,2,…, *K*/2, *P*[0] = 0 for  *k* = *K*, *K* + 1,…, *U* = (*KM* − 2)/2.

When polyphase filtering structures are used with the FFT and IFFT pairs to implement the FBMC/OQAM system, the synthesis polyphase filter at the transmitter is defined as

(9) $$\begin{array}{c} a_{k}\left[m\right]=p\left[k+mM\right]. \end{array}$$

At the receiver, the analysis filter polyphase is given by

(10) $$\begin{array}{c} b_{k}\left[m\right]=a_{M-1-k}\left[m\right]=p\left[M-1-k+mM\right]. \end{array}$$

The transmitted FBMC/OQAM signal *y*[*m*] in (1) is assumed to go through a time-varying frequency-selective Rayleigh fading channel with background additive white Gaussian noise (AWGN). Assuming that the allocated bandwidth for each subcarrier is less than the coherence bandwidth of the wireless channel, each subcarrier undergoes frequency nonselective flat-fading. Thus, the received signal sample over the *k*th subcarrier for the *m*th FBMC/OQAM symbol can be expressed as

(11) $$\begin{array}{c} r_{k}\left[m\right]=y_{k}\left[m\right]h_{k}\left[m\right]+w_{k}\left[m\right], \end{array}$$

where *h*_*k*_[*m*] is a complex valued fading process over the *k*th subcarrier for the *m*th FBMC/OQAM symbol and *w*_*k*_[*m*] is an additive white Gaussian noise (AWGN) process. The noise processes {*w*_*k*_[*m*]}_*k*=0,1,…,*M*−1_ are assumed to be mutually independent and identically distributed zero-mean complex Gaussian processes, with equal variances *σ*_*w*_^2^. The fading process *h*_*k*_[*m*] = |*h*_*k*_[*m*]|*e* ^−*jφ*_*k*_(*m*)^ is assumed to be a zero-mean complex Gaussian process with uniformly distributed phase *φ*_*k*_(*m*) on [0,2*π*] and with Rayleigh distributed envelope |*h*_*k*_[*m*]|. The variances of the fading processes {*h*_*k*_[*m*]}_*k*=0,1,…,*M*−1_ are all assumed to be equal to *σ*_*h*_^2^.

After processing the received signal *r*_*k*_[*m*] with the analysis filter bank block, the resulting signal at the input of channel estimation and equalization is given by

(12) sk[n]=[rk[m]∗fk[m]]↓M/2=xk[n]·qk[n]+ηk[n],

where *q*_*k*_[*n*] = [*h*_*k*_[*m*]*g*_*k*_[*m*]∗*f*_*k*_[*m*]]_↓*M*/2_ with ∗ denoting the convolution operator and *η*_*k*_[*n*] being a Gaussian noise process with variance *σ*_*η*_^2^. Under the assumption of nearly perfect reconstruction (NPR) conditions of the prototype filter, it follows that *q*_*k*_[*n*] = [*h*_*k*_[*m*]*g*_*k*_[*m*]∗*f*_*k*_[*m*]]_↓*M*/2_≅*h*_*k*_[*n*]. Thus, (12) is reduced to

(13) $$\begin{array}{c} s_{k}\left[n\right]=x_{k}\left[n\right]h_{k}\left[n\right]+\eta_{k}\left[n\right],\;k=0,1,\ldots ,M-1. \end{array}$$

The statistical properties of the fading process *h*_*k*_[*n*] are given by its power spectrum density (PSD) and autocorrelation function (ACF). Indeed, the PSD of *h*_*k*_[*n*] is defined by the well-known U-shaped band-limited Jakes spectrum with maximum Doppler frequency *f*_*d*_ [30]:

(14) $$\begin{array}{c} S\left[f\right]=\{\begin{array}{cc} \frac{1}{\pi f_{d}\sqrt{{1-\left(f/f_{d}\right)}^{2}}}, & \left|f\right|\leq f_{d}\\ 0, & \text{elsewhere}, \end{array} \end{array}$$

where *f*_*d*_ = *v*/*λ* with *v* is the mobile speed and *λ* is the wave length of the carrier wave. The corresponding normalized discrete-time autocorrelation function (ACF) hence satisfies

(15) $$\begin{array}{c} R_{k}=J_{0}\left(2\pi f_{d}T_{s}\left|k\right|\right), \end{array}$$

where *J*_0_(·) is the zero-order Bessel function of the first kind, *T*_*s*_ is the symbol period, and *f*_*d*_ *T*_*s*_ denotes the Doppler rate.

To exploit the statistical properties of the fading channel given by its PSD and ACF, the fading process *h*_*k*_[*n*] can be modeled by a *p*th order AR process, denoted by AR(*p*) and defined as follows [31]:

(16) $$\begin{array}{c} h_{k}\left[n\right]=-\overset{p}{\underset{i=1}{\sum }}a_{i}h_{k-i}\left[n\right]+\upsilon_{k}\left[n\right], \end{array}$$

where {*a*_*i*_}_*i*=1,2,…,*p*_ are the AR model parameters and *υ*_*k*_[*n*] denotes the zero-mean complex white Gaussian driving process with equal variance *σ*_*υ*_^2^ over all subcarriers. Increasing the AR mode order *p* will provide better fitting between the resulting model and the theoretical PSD and ACF [11, 31]. However, the computational complexity of the resulting channel estimation algorithm will also increase. Therefore, a compromise between the accuracy of the model and the computational complexity of the estimation algorithm has to be found. When the Doppler rate *f*_*d*_ *T*_*s*_ is available at the receiver, the AR parameters {*a*_*i*_}_*i*=1,2,…,*p*_ can be computed by solving the so-called Yule-Walker (YW) equations. However, as the Doppler rate *f*_*d*_ *T*_*s*_ is usually unknown, we propose to complete the joint estimation of the fading process *h*_*k*_[*n*] and its AR parameters {*a*_*i*_}_*i*=1,2,…,*p*_ based on dual Kalman filters.

## 3. Dual Kalman Filters Based Channel Estimation

The estimation of the fading process *h*_*k*_[*n*] along the *n*th FBMC/OQAM symbol will be performed in two steps. Firstly, the fading process *h*_*k*_[*n*] at the pilot symbol position is estimated using dual Kalman filters. Secondly, the fading process at data symbol position will then be estimated by using some interpolation methods such as linear, spline, or low-pass interpolation.

**Figure 3:**
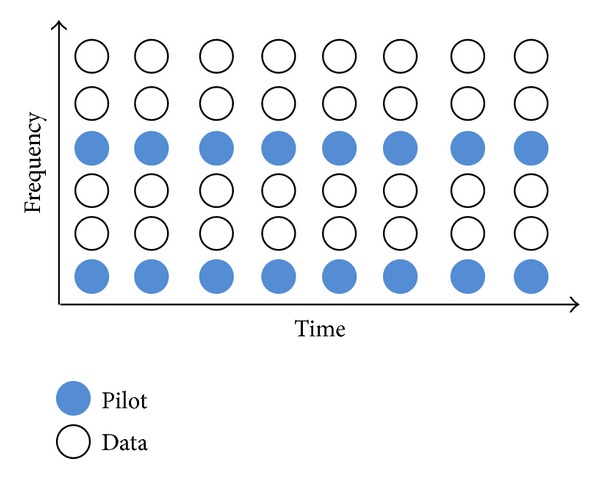
Comb-type pilot arrangement.

When using comb-type pilot arrangement [8] as shown in Figure 3, *N*_*p*_ pilots are uniformly inserted into *x*_*k*_[*n*] as follows:

(17) xk[n]=xuL+i[n]={xuL,pilot[n],i=0xuL+i,data[n],i=1,2…,L−1,

where *x*_*uL*,pilot_[*n*] is the *u*th pilot symbol with *u* = 0,1,…, *N*_*p*_ − 1, and *L* = *M*/*N*_*p*_ is the pilot interval spacing with *M* being the number of subcarriers.

**Figure 4:**
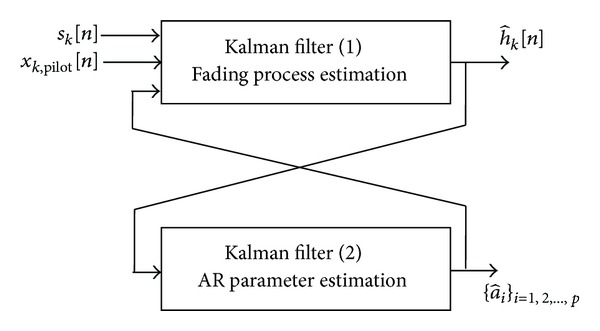
Dual Kalman filters based structure for the joint estimation of the fading process and its AR parameters over the *n*th FBMC/OQAM symbol.

Thus, using known comb-type pilot symbols *x*_*k*_[*n*] = *x*_*k*,pilot_[*n*], we propose to jointly estimate the fading process *h*_*k*_[*n*] and its AR parameters {*a*_*i*_}_*i*=1,2,…,*p*_ based on dual Kalman filters as shown in Figure 4. Indeed, the first Kalman filter in Figure 4 uses the pilot symbol *x*_*k*,pilot_[*n*], the output of the analysis filter bank *s*_*k*_[*n*], and the latest estimated AR parameters ${\left\{\hat{a}{}_{i}\right\}}_{i=1,2,\ldots ,p}$ to estimate the fading process *h*_*k*_[*n*], while the second Kalman filter uses the estimated fading process $\hat{h}{}_{k}\left[n\right]$ to update the AR parameters.

### 3.1. Fading Process Estimation at Pilot Symbol Positions

In this subsection, our purpose is to estimate the fading process *h*_*k*_[*n*] over the *n*th FBMC/OQAM symbol based on Kalman filtering with known pilot symbols. To this end, let us define the state vector as follows:

(18) $$\begin{array}{c} h_{k}={\left[\begin{array}{cccc} h_{k} & h_{k-1} & \cdots & h_{k-p+1} \end{array}\right]}^{T}. \end{array}$$

Note that, for the sake of simplicity and clarity of presentation, the time index subscript is dropped. Then, (16) can be written in the following state-space form:

(19) $$\begin{array}{c} h_{k}=\phi h_{k-1}+g\upsilon_{k}, \end{array}$$

where

(20) $$\begin{array}{c} \phi =\left[\begin{array}{cccc} {-a}_{1} & {-a}_{2} & \cdots & {-a}_{p}\\ 1 & 0 & \cdots & 0\\ \vdots & \vdots & \ddots & \vdots \\ 0 & \cdots & 1 & 0 \end{array}\right],\\ g={\left[\begin{array}{cccc} 1 & 0 & \cdots & 0 \end{array}\right]}^{T}. \end{array}$$

In addition, given (13) and (18), it follows that

(21) $$\begin{array}{c} s_{k}=x_{k}^{T}h_{k}+\eta_{k}, \end{array}$$

where $x_{k}={\left[\begin{array}{cccc} x_{k,\text{pilot}} & 0 & \cdots & 0 \end{array}\right]}^{T}$.

Hence, (19) and (21) represent the state-space model dedicated to the *n*th FBMC/OQAM symbol fading channel system (13) and (16). A standard Kalman filtering algorithm can then be carried out to provide the estimation $\hat{h}{}_{k/k}$ of the state vector **h**_*k*_ given the set of observations {*s*_*i*_}_*i*=1,…,*k*_. To this end, let us introduce the so-called innovation process *α*_*k*_ which can be obtained as follows:

(22) $$\begin{array}{c} \alpha_{k}=s_{k}-x_{k}^{T}\phi \hat{h}{}_{k-1/k-1}. \end{array}$$

The variance of the innovation process can be expressed as

(23) $$\begin{array}{c} C_{k}=E\left[\alpha_{k}\alpha_{k}^{*}\right]=x_{k}^{T}P_{k/k-1}x_{k}+\sigma_{\eta_{k}}^{2}, \end{array}$$

where the so-called *a priori* error covariance matrix **P**_*k*/*k*−1_ can be recursively obtained as follows:

(24) $$\begin{array}{c} P_{k/k-1}=\phi P_{k/k-1}\phi^{H}+g\sigma_{\upsilon }^{2}g^{T}. \end{array}$$

The Kalman gain is calculated in the following manner:

(25) $$\begin{array}{c} K_{k}=P_{k/k-1}x_{k}C_{k}^{-1}. \end{array}$$

The *a posteriori* estimate of the state vector and the fading process are, respectively, given by

(26) $$\begin{array}{c} \hat{h}{}_{k/k}=\phi \hat{h}{}_{k-1/k-1}+K_{k}\alpha_{k},\\ \hat{h}{}_{k}=\hat{h}{}_{k/k}=g^{T}\hat{h}{}_{k/k}. \end{array}$$

The error covariance matrix is updated as follows:

(27) $$\begin{array}{c} P_{k/k}=P_{k/k-1}-K_{k}x_{k}^{T}P_{k/k-1}. \end{array}$$

It should be noted that the state vector and the error covariance matrix are initially assigned to zero vector and identity matrix, respectively; that is, $\hat{h}{}_{0/0}=0$ and **P**_0/0_ = **I**_*p*_.

Equations (22)–(27) can be evaluated providing the availability of the AR parameters that are involved in the transition matrix **ϕ** and the driving process variance *σ*_*υ*_^2^. They will be estimated in the next subsections.

### 3.2. Estimation of the AR Parameters

To estimate the AR parameters from the estimated fading process $\hat{h}{}_{k}$, (26) are firstly combined such as the estimated fading process is a function of the AR parameters:

(28) h^k=gTϕh^k−1+gTKkαk=h^k−1Tak+ςk,

where $\hat{h}{}_{k-1}=\left[\begin{array}{cccc} \hat{h}{}_{k-1} & \hat{h}{}_{k-2} & \cdots & \hat{h}{}_{k-p} \end{array}\right]$ and $a_{k}={\left[\begin{array}{cccc} {-a}_{1} & {-a}_{2} & \cdots & {-a}_{p} \end{array}\right]}^{T}$. In addition, the variance of the process *ς*_*k*_ = **g** ^*T*^ **K**_*k*_ *α*_*k*_ is given by

(29) $$\begin{array}{c} \sigma_{\varsigma_{k}}^{2}=g^{T}K_{k}C_{k}K_{k}^{H}g. \end{array}$$

When the channel is assumed stationary, the AR parameters are invariant and satisfy the following relationship:

(30) $$\begin{array}{c} a_{k}=a_{k-1}. \end{array}$$

As (28) and (30) define a state-space representation for the estimation of the AR parameters, a second Kalman filter can be used to recursively estimate **a**_*k*_ as follows:

(31) $$\begin{array}{c} \hat{a}{}_{k}=\hat{a}{}_{k-1}+K_{a_{k}}\left(\hat{h}{}_{k}-\hat{h}{}_{k-1}^{T}\hat{a}{}_{k-1}\right), \end{array}$$

where the Kalman gain **K**_**a**_*k*__ and the update of the error covariance matrix **P**_**a**_ are, respectively, given by

(32) $$\begin{array}{c} K_{a_{k}}=P_{a_{k-1}}{\hat{h}{}_{k-1}^{*}\left(\hat{h}{}_{k-1}^{H}P_{a_{k-1}}\hat{h}{}_{k-1}+\sigma_{\varsigma_{k}}^{2}\right)}^{-1},\\ P_{a_{k}}=P_{a_{k-1}}-K_{a_{k}}\hat{h}{}_{k-1}^{T}P_{a_{k-1}} \end{array}$$

with initial conditions $\hat{a}{}_{0}=0$ and **P**_**a**_0__ = **I**_*p*_.

### 3.3. Estimation of the Driving Process Variance

To estimate the driving process variance *σ*_*υ*_^2^, the Riccati equation is first obtained by inserting (24) in (27) as follows:

(33) $$\begin{array}{c} P_{k/k}=\phi P_{k-1/k-1}\phi^{H}+g\sigma_{\upsilon_{k\;}}^{2}g^{T}-K_{k}x_{k}^{T}P_{k/k-1}. \end{array}$$

Taking into account that **P**_*k*/*k*−1_ is a symmetric Hermitian matrix, one can rewrite (25) in the following manner:

(34) $$\begin{array}{c} b_{k}^{T}P_{k/k-1}=C_{k}K_{k}^{H}, \end{array}$$

By combining (33) and (34), *σ*_*υ*_^2^ can be expressed as follows:

(35) $$\begin{array}{c} \sigma_{\upsilon }^{2}=f\left[P_{k/k}-\phi P_{k-1/k-1}\phi^{H}+K_{k}C_{k}K_{k}^{H}\right]f^{T}, \end{array}$$

where **f** = [**g** ^*T*^ **g**]^−1^ **g** ^*T*^ = **g** ^*T*^ is the pseudoinverse of **g**.

Thus, we propose to estimate *σ*_*υ*_^2^ recursively as follows:

(36) σ^υk2=λσυk−12+[1−λ] ×f[Pk/k−ϕPk−1/k−1ϕH+Kk|αk|2KkH]fT,

where the variance of the innovation process *C*_*k*_ is replaced by its instantaneous value |*α*_*k*_|^2^ and *λ* is the forgetting factor. It should be noted that *λ* can be either constant or time-varying (e.g., *λ*_*k*_ = (*k* − 1)/*k*).

### 3.4. Fading Process Estimation at Data Symbol Positions

Once the fading process at pilot symbol positions is estimated by the dual Kalman filters, the fading process at data symbol positions will then be estimated by using some interpolation methods such as linear, spline, or low-pass interpolation [8].

*(1) Linear Interpolation.* Using linear interpolation method, the channel estimates at data positions, *uL* < *k* < (*u* + 1)*L*, are given by

(37) h^k=(h^pk+1−h^pk)lL+h^pk, 0≤l<L,

where $\hat{h}{}_{p_{k}}$ is the estimated fading process at pilot symbol position.

*(2) Low-Pass Interpolation.* The low-pass interpolation is carried out by inserting zeros into the data symbol positions of the original sequence and then applying a special low-pass filter. In this paper, we use the Matlab function *interp.*

*(3) Spline Interpolation.* Spline interpolation produces a smooth and continuous polynomial fitted to the estimated fading process at pilot symbol positions. In this paper, we use the Matlab function *spline.*

### 3.5. Fading Channel Equalization

Once the fading process at pilot and data symbols is estimated using the proposed approach, channel equalization can be performed by multiplying (13) with a normalized version of the complex conjugate of the channel estimate as follows:

(38) $$\begin{array}{c} \hat{x}{}_{k}\left[n\right]=s_{k}\left[n\right]\left(\frac{\hat{h}{}_{k}^{*}\left[n\right]}{{\left|\hat{h}{}_{k}[n]\right|}^{2}}\right). \end{array}$$

## 4. Simulation Results

In this section, we carry out a comparative simulation study on the estimation of FBMC/OQAM fading channels between the proposed dual Kalman filters based channel estimator and the conventional LMS and RLS channel estimators. In addition, we test the performance of three interpolation techniques, namely, low-pass, spline, and linear interpolation. Furthermore, we investigate the effect of the number of pilot symbols *N*_*p*_ and Doppler rate *f*_*d*_ *T*_*s*_ on the BER performance.

In all of our simulations, the fading channels are generated according to the autoregressive based method presented in [31]. The autoregressive model order *p* in the proposed dual Kalman filters based estimator is set to *p* = 2. The step size for LMS algorithm is set to *μ* = 0.5 and the forgetting factor for RLS algorithm is set to *λ* = 0.1.

**Figure 5:**
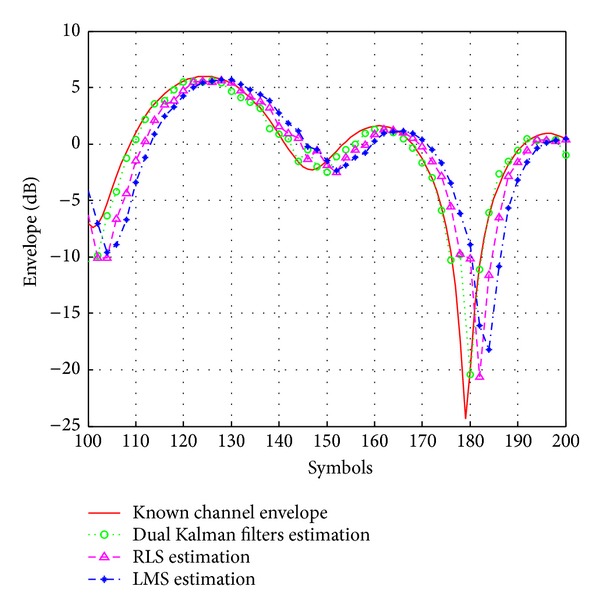
Envelope of estimated fading process using the various estimators. SNR = 15 dB, *M* = 2048, *N*_*p*_ = 1024, and *f*_*d*_ *T*_*s*_ = 0.0167.

Figure 5 shows the envelope of estimated fading process using the various channel estimators with low-pass interpolation, SNR = 15 dB, *M* = 2048, *N*_*p*_ = 1024, and*f*_*d*_ *T*_*s*_ = 0.0167. One can notice that the dual Kalman filters based estimator provides much better estimation than the LMS and RLS based ones.

**Figure 6:**
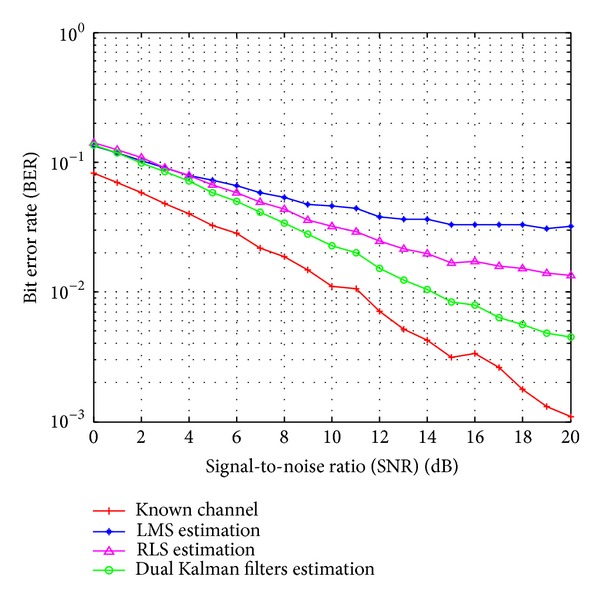
BER performance versus SNR for the FBMC/OQAM system with the various channel estimators. *M* = 2048, *N*_*p*_ = 1024, and *f*_*d*_ *T*_*s*_ = 0.0167.

**Figure 7:**
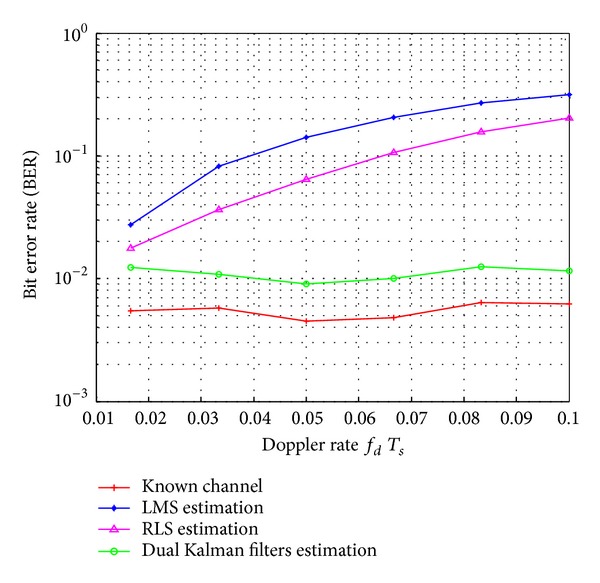
BER performance versus Doppler rate for the FBMC/OQAM system with the various channel estimators. SNR = 15 dB, *M* = 2048, and *N*_*p*_ = 1024.

Figure 6 shows the BER performance versus SNR for the FBMC/OQAM system when using the various channel estimators with *M* = 2048, *N*_*p*_ = 1024 pilots, *f*_*d*_ *T*_*s*_ = 0.0167, and using low-pass interpolation. According to Figure 6, the proposed dual Kalman filters based estimator outperforms the conventional LMS and RLS estimators with the performance difference increasing as the SNR increases.  Figure 7 displays the BER performance of the FBMC/OQAM system versus Doppler rate when using the various channel estimators with low-pass interpolation, *M* = 2048, and *N*_*p*_ = 1024 pilots. One can notice that the dual Kalman filters based estimator yields lower BER performance than the LMS and RLS estimators with the BER performance difference increasing as the Doppler rate increases. This confirms that the dual Kalman filters based estimator can exploit the fading channel statistics and can track fast variations of rapidly varying fading channels.

**Figure 8:**
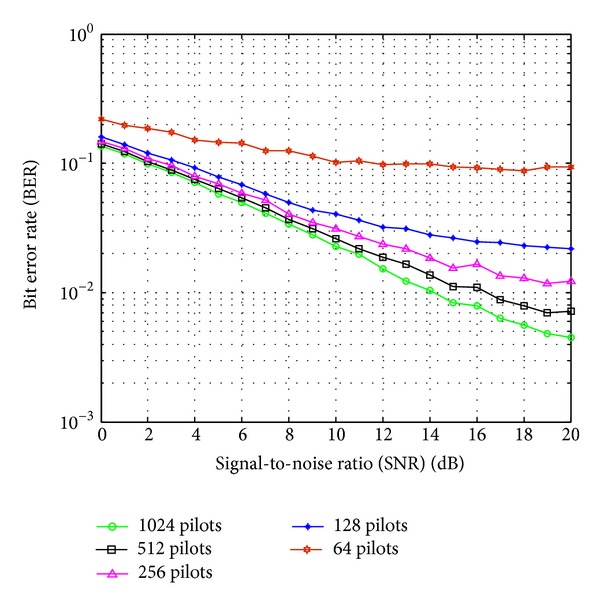
BER performance versus SNR for the FBMC/OQAM system with different number of pilot symbols. *M* = 2048 and *f*_*d*_ *T*_*s*_ = 0.0167.

The effect of changing the number of pilot symbols *N*_*p*_ on the BER performance of the FBMC/OQAM system when using dual Kalman filters based channel estimator with low-pass interpolation is shown in Figure 8. Indeed, increasing the number of pilot symbols *N*_*p*_ will improve the BER performance of the system.

**Figure 9:**
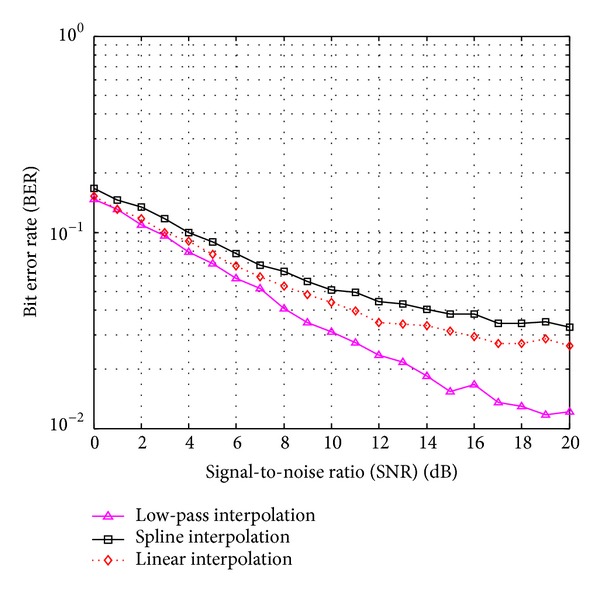
BER performance versus SNR for the FBMC/OQAM system with the various interpolation methods. *M* = 2048, *N*_*p*_ = 256, and *f*_*d*_ *T*_*s*_ = 0.0167.

Figure 9 shows the BER performance of the FBMC/OQAM system when using dual Kalman filters with the various interpolation methods, *M* = 2048, *N*_*p*_ = 256 pilots, and *f*_*d*_ *T*_*s*_ = 0.0167. It is confirmed that the low-pass interpolation yields the best BER performance.

## 5. Conclusions

This paper addresses the estimation and equalization of FBMC/OQAM fading channels based on pilot symbols. A dual Kalman filters based structure is proposed for the joint estimation of the fading process and its AR parameters over each subcarrier at pilot symbol positions. The fading process at data symbol positions is then obtained by using some interpolation methods such as linear, spline, or low-pass interpolation. The simulation results showed that the proposed dual Kalman filters based estimator outperforms the LMS and RLS based ones. In addition, the low-pass interpolation is confirmed to outperform both spline and linear interpolation.
